# Mothers’ home management practices during febrile seizures in children under six years of age in Jordan

**DOI:** 10.1371/journal.pone.0355159

**Published:** 2026-08-07

**Authors:** Maha Atout, Nouraldun M. Alhsenat, Huda Gharaibeh, Fatimah S. Tarawneh, Atheer Qashou

**Affiliations:** 1 Faculty of Nursing, Philadelphia University, Amman, Jordan; 2 Princess Muna College of Nursing, Mutah University, Al-Karak, Jordan; 3 Maternal-Child Health and Midwifery Department, Faculty of Nursing, Jordan University of Science and Technology, Irbid, Jordan; 4 Princess Muna College of Nursing, Mutah University, Al-Karak, Jordan; 5 Arab American University, Jenin, OPT; Kalinga Institute of Medical Sciences, INDIA

## Abstract

**Background:**

Febrile seizure is the most prevalent seizure disorder among young children, and it commonly occurs in the home setting, where mothers are often the first responders. First-aid management of febrile seizures is also important to avoid complications and help parents feel less anxious. Nevertheless, there is a paucity of evidence describing the home-management practices of mothers in Jordan.

**Objective:**

The purpose of this study was to describe the home-management practices of mothers of children under six years of age with febrile seizures in Jordan and to determine factors associated with appropriate and inappropriate practices.

**Methods:**

A cross-sectional descriptive survey design was used. Data were collected between August and December 2021 from mothers attending pediatric outpatient clinics and pediatric wards in two public hospitals in northern Jordan. A structured questionnaire developed by Elbilgahy and Abd El Aziz (2018) was used to assess mothers’ home-management practices. Predictors of mothers’ home-management practices were analyzed using SPSS version 26, descriptive statistics, Pearson correlation analysis, and backward multiple regression analysis.

**Results:**

In total, 68.4% of the mothers exhibited proper home-management behaviors in the course of febrile seizure. The majority of mothers reported that they put the child in a safe position (86.9%), noticed the manifestations and duration of seizures (84.5%), and tried to decrease body temperature (85.7%). Nevertheless, a considerable percentage of them were panic (61.9%) and doing the wrong things like cardiac massage (23.8%). Regression analysis showed that higher maternal knowledge and more positive attitudes were significantly associated with better home-management practice scores, which explained 27% of the variance of home-management practices among mothers.

**Conclusion:**

Despite the fact that over two-thirds of mothers showed proper actions, there were still gaps in some essential first-aid actions in case of febrile seizures. There were a number of factors that were linked to home-management practices by mothers, which underscored the need to enhance the education of caregivers about the first-aid management of febrile seizures. Specific educational programs by healthcare providers are required to enhance the response of caregivers and encourage safe home management of febrile seizures among children.

## Introduction

Febrile seizures (FS) are the most prevalent seizure disorder in early childhood, occurring in approximately 2–5% of children under six years of age worldwide. They are associated with fever in the absence of central nervous system infection, metabolic imbalance, or previous afebrile seizures [1]. Febrile seizures are generally categorized as either simple or complex, with important implications for clinical assessment, parental counseling, and follow-up care [2]. Caregivers’ ability to monitor seizure duration, recurrence during the same febrile illness, and focal features is vital, as prolonged or recurrent seizures may require immediate medical attention.

Caregivers play an important role in keeping the home safe during febrile seizures for home management. If seizures persist for more than five minutes, they are unlikely to stop spontaneously and may require emergency treatment or rescue medication (if available) [1,3]. Therefore, the caregiver’s ability to recognize a seizure, provide appropriate first aid, and seek prompt medical attention is crucial in preventing secondary injury and ensuring timely medical care.

Children who have a normal neurological examination and function normally after a febrile seizure have no indication for routine neurodiagnostic testing, including EEG or neuroimaging [4]. Rather, management should be directed at the underlying cause of the fever and serious infections be ruled out when indicated. These recommendations emphasize the importance of providing caregivers with clear, evidence-based instructions on maintaining safety at home and reassure them that febrile seizures are usually benign.

Although the above recommendations, there is still a lack of knowledge among caregivers about the recurrence of a fever and febrile seizure. There is evidence that antipyretic drugs improve the child’s comfort but are unreliable in preventing the recurrence of febrile seizures during different febrile illnesses [1,5]. While some studies have suggested there could be a decrease in recurrence in the same febrile period, there is little evidence of a preventive effect on later episodes [6]. This is clinically significant as education of caregivers should include appropriate first-aid measures and warning signs, not aggressive fever management measures.

Counselling for caregivers is considered one of the pillars of febrile seizure management. Recent European consensus recommendations underline the need to provide families with standardized information on seizure safety precautions and the risk of recurrence, but also information regarding signs that demand urgent medical attention to avoid anxiety-driven and potentially unsafe behaviors [7]. However, there is evidence that the recommendations provided by healthcare professionals are not always consistent with current best-practice guidelines. For instance, in Greece a nationwide survey of pediatricians revealed that there was a widespread use of aggressive treatment for fevers even though there was limited evidence to support this, and there were significant differences in the education of the caregivers [8].

Misconceptions about childhood fever (fever phobia) still affect how caregivers manage febrile illnesses and febrile seizures [9,10]. These misconceptions can lead to anxiety-related behaviours such as unnecessary visits to the ED, unsafe cooling practices and inappropriate seizure first aid. International studies have also demonstrated significant relationships between caregivers’ fear and uncertainty and inappropriate management intentions during febrile seizure episodes [11].

Home-management practices during febrile seizure episodes may be affected by socio-cultural differences and healthcare systems. Qualitative data from Ghana, for instance, revealed traditional remedies being used and the delay in seeking medical attention during a seizure, suggesting the need for context-specific assessments of caregiver behaviours [12]. These considerations must also be understood to design culturally appropriate educational approaches.

In Jordan, earlier research has found low awareness of febrile seizures in mothers, and a high correlation between caregiver anxiety and uncertainty in relation to limited awareness of febrile seizures [13]. Other studies have suggested that Jordanian parents still have incorrect perceptions about the treatment of fever, including early antipyretic drugs administration and dosage [14]. However, in the light of these results, little is known about the actual home-management of mothers during a febrile seizure episode in Jordan.

Further studies of maternal home-management practices are necessary to guide the development of targeted practices of education and standardized counseling approaches to better address caregivers’ responses during seizure episodes and to determine the effects on preventing complications and child safety. Accordingly, the purpose of this study was to describe the home-management practices of mothers of children under six years of age with febrile seizures in Jordan and to identify factors associated with appropriate and inappropriate practices.

## Methods

### Study design

A cross-sectional descriptive survey design was used to assess mothers’ home-management practices during febrile seizure episodes in children under six years of age. This design was appropriate because it enabled the collection of data at a single point in time to describe mothers’ practices and identify factors associated with appropriate and inappropriate responses to febrile seizures. However, the present study focused specifically on mothers’ home-management practices. This study followed the Strengthening the Reporting of Observational Studies in Epidemiology (STROBE) guidelines for cross-sectional studies.

### Study setting

The study was conducted in the pediatric outpatient clinics and pediatric wards of two major public hospitals in northern Jordan: Princess Haya Military Hospital (PHMH) and Princess Rahma Teaching Hospital (PRTH). These hospitals are equipped with both curative and preventive services for children and serve a large population from nearby rural and urban areas. Most families attending these hospitals are from low- to middle-income backgrounds, making these hospitals suitable settings for studying mothers’ home-management practices.

### Sample and sampling procedure

Following an ethical approval granted by the Jordan University of Science and Technology (Approval No: 2021/333), the participants were recruited and data were collected from 10 August 2021 until 31 December 2021. A convenient sampling method was utilized. Eligible participants were mothers whose children were younger than six years of age, had experienced at least one febrile seizure, and attended the outpatient clinics or accompanied their children during admission to the pediatric wards at the selected hospitals.

Mothers were excluded if their children had seizures related to metabolic disorders, neurological disorders, or other non-febrile causes. Based on the recommendations of Cohen for multiple regression analysis, the sample size was calculated at N = 84 participants assuming a medium effect size, significance level of 0.05 and statistical power of 0.80. The total number of mothers recruited is 84.

### Instrument

The data were gathered by a structured questionnaire adopted by Elbilgahy and Abd El Aziz [15]. Since the original questionnaire was written in Arabic, there was no need for language translation. Experts in pediatric nursing and maternal-child health reviewed the instrument and were asked to provide feedback on its clarity, content validity and cultural appropriateness for the Jordanian mothers. A few spelling corrections were made as needed to enhance understanding without altering the meaning of the items. This questionnaire comprised four sections, which included questions on demographic characteristics, knowledge, attitudes and home-management practices. Even if this study emphasized the mothers’ home-management practices, their scores in knowledge and attitude were also entered as explanatory variables in the regression analysis. Detailed analyses of the knowledge and attitude domains from the same cohort have been reported previously [16].

The knowledge domain consisted of 12 dichotomous (correct/incorrect) questions that assessed mothers’ knowledge about febrile seizures, their definition, signs and symptoms, their recurrence, prevention, treatment, and first-aid management. Correct answers were assigned one point, whereas incorrect answers received zero points, with higher scores indicating greater knowledge. In the present study, the knowledge scale demonstrated good internal consistency (Cronbach’s α = 0.86).

The attitude domain comprised 8 items rated on a five-point Likert scale to assess mothers’ attitudes toward febrile seizure management. Higher scores indicated more positive attitudes. In the present study, Cronbach’s α was 0.86, indicating good internal consistency of the attitude scale.

The home-management practice domain comprised 12 dichotomous (Yes/No) items that evaluated mothers’ first-aid practices during febrile seizure episodes. Correct practices were assigned one point, whereas incorrect practices received zero points, producing a total score ranging from 0 to 12. Consistent with the original instrument, scores ≥60% were classified as indicating appropriate practice, whereas scores <60% indicated inappropriate practice. Unsafe practices were reverse-coded so that higher scores reflected safer home-management practices. The practice scale demonstrated good internal consistency in the present study (Cronbach’s α = 0.81).

### Data collection procedure

Eligible mothers were approached during their visit to the pediatric outpatient clinics or while accompanying their hospitalized children in the pediatric wards. The researcher explained the purpose of the study, assured participants that participation was voluntary, and confirmed that refusal to participate would not affect the care provided to their children. Participants who agreed to take part in the study provided written informed consent before data collection. Data were collected through face-to-face structured interviews conducted by the researcher and two trained assistants in the waiting areas of the outpatient clinics or pediatric wards. Each interview lasted approximately 5–10 minutes. The questionnaires were administered in Arabic.

### Data analysis

Data were analyzed using the Statistical Package for the Social Sciences (SPSS), version 26. Descriptive statistics, including frequencies, percentages, means, and standard deviations, were used to summarize the demographic characteristics and mothers’ home-management practices.

The dependent variable in this study was the mothers’ home-management practice score. Independent variables included maternal demographic characteristics, seizure-related child characteristics, and mothers’ knowledge and attitude scores. Pearson correlation analysis was performed to examine the relationships between demographic variables and mothers’ practice scores. Before the regression analysis, the assumptions of linearity, independence of errors, homoscedasticity, normality of residuals, and the absence of multicollinearity were assessed. Backward multiple regression analysis was conducted to identify predictors of mothers’ home-management practices during febrile seizure episodes. This approach was selected because it allows all candidate predictor variables to be entered initially and then sequentially removes non-significant variables, resulting in a parsimonious model while accounting for the potential influence of all measured covariates. Statistical significance was set at p ≤ 0.05.

### Ethical considerations

Ethical approval for this study was obtained from the Institutional Review Board of Jordan University of Science and Technology (Reference No. 2021/333; approved on 05 August 2021). Additional permissions were obtained from the Royal Medical Services and the Ministry of Health to conduct the study at Princess Haya Military Hospital and Princess Rahma Teaching Hospital. Written informed consent was obtained from all participants before participation in the interview, and the confidentiality of participants’ information was strictly maintained throughout the study.

## Results

### Sociodemographic characteristics of mothers

A total of 84 mothers participated in the study. Their mean age was 29.89 ± 5.66 years (range: 18–41 years), and half (50.0%) had a diploma or higher level of education. The remaining demographic characteristics are presented in Table 1.

**Table 1:** Demographic characteristics of the participants (N = 84).

Variables	Frequency	%
**Mothers’ education**		
Less than secondary education	10	11.9
Secondary education	32	38.1
Diploma and more	42	50.0
**Mothers’ age in years**		
Mean ± SD	29.89 ± 5.66	
Minimum	18	
Maximum	41	

### Sociodemographic characteristics of children

The mean age of the children was 24.55 ± 15.41 months (range: 3–72 months), and 60.7% were male. The mean age at the first occurrence of a febrile seizure was 19.54 ± 12.54 months. Most children (61.9%) had experienced one febrile seizure episode, whereas 16.7% had experienced two episodes and 21.4% had experienced more than two episodes.

Half of the seizures (50.0%) lasted less than five minutes, whereas 27.4% lasted between 6 and 15 minutes, and 22.6% lasted longer than 15 minutes. Cyanosis during febrile seizure episodes was reported in 73.8% of the children. The majority of mothers (88.1%) reported having only one child affected by febrile seizures. The detailed characteristics of the children are summarized in Table 2

**Table 2:** Sociodemographic characteristics of the children sample (N = 84).

Variables	Frequency	%
**Gender of child**		
Male	51	60.7
Female	33	39.3
**The child’s age at the first occurrence of febrile seizure (in months)**		
Mean ± SD	19.54 ± 12.54	
Minimum	1	
Maximum	66	
**Number of children who suffer from a febrile seizure in the family**		
One	74	88.1
Two	9	10.7
Four	1	1.2
**Numbers of febrile seizures from birth**		
Once	52	61.9
Twice	14	16.7
More than twice	18	21.4
**Duration of febrile seizure**		
Less than 5 minutes	42	50.0
From 6 minutes to 15 minutes	23	27.4
More than 15 minutes	19	22.6
**Cyanosis during the febrile seizure**		
Yes	62	73.8
No	22	26.2
**Child age in months**		
Mean ± SD	24.55 ± 15.41	
Minimum	3	
Maximum	72	

### Mothers’ immediate responses to febrile seizure episodes

Regarding mothers’ immediate responses during febrile seizure episodes, more than half (59.5%) reported shouting and calling for help. Approximately one-third (33.3%) reported taking their child directly to the emergency department, whereas smaller proportions reported visiting a private clinic (3.6%) or seeking assistance from neighbors (3.6%) (Table 3).

**Table 3:** Immediate mothers’ actions when the attack of febrile seizure occurs.

Immediate response	n (%)
Shouted and called for help	50 (59.5)
Went directly to the emergency department	28 (33.3)
Other responses (private clinic or neighbour)	6 (7.2)

### Mothers’ home-management practices during febrile seizures

Mothers’ home-management practices were assessed using a 12-item practice scale, with scores ranging from 0 to 12. Based on the instrument scoring criteria, scores ≥60% indicated appropriate practice.

Most mothers demonstrated appropriate practices in several key first-aid measures. Specifically, 95.2% reported taking their child to a doctor or hospital, 86.9% placed the child in a safe position, 85.7% attempted to reduce the child’s body temperature, and 84.5% reported observing the seizure manifestations and duration.

However, several inappropriate responses were also reported. A substantial proportion of mothers reported panicking during seizure episodes (61.9%), performing cardiac massage (23.8%), and shaking the child during seizures (34.5%). In addition, 32.1% reported placing objects in the child’s mouth during seizures.

Overall, there were 689 correct responses out of 1,008 possible responses, indicating that 68.4% of mothers demonstrated appropriate home-management practices during febrile seizure episodes (Table 4).

**Table 4:** Mothers’ practices of febrile seizure.

Variables	Frequency	%
**Take the child to a doctor or the hospital**		
Correct	80	95.2
Incorrect	4	4.8
**Decrease the child’s body temperature**		
Correct	72	85.7
Incorrect	12	14.3
**Put the child in a soft and safe place**		
Correct	73	86.9
Incorrect	11	13.1
**Observe seizure manifestations and duration**		
Correct	71	84.5
Incorrect	13	15.5
**Perform mouth-to-mouth resuscitation**		
Correct	74	88.1
Incorrect	10	11.9
**Keep calm**		
Correct	32	38.1
Incorrect	52	61.9
**Apply cardiac massage**		
Correct	64	76.2
Incorrect	20	23.8
**Put the child on his/her side**		
Correct	57	67.9
Incorrect	27	32.1
**Shake and rouse the seizure child**		
Correct	29	34.5
Incorrect	55	65.5
**Put something in his/her mouth**		
Correct	57	67.9
Incorrect	27	32.1
**Remove secretion from the child’s nose and mouth**		
Correct	33	39.3
Incorrect	51	60.7
**Restrain the seizure child**		
Correct	47	56
Incorrect	37	44

### Predictors of mothers’ home-management practices

Backward multiple regression analysis was performed to identify predictors of mothers’ home-management practice scores. Before conducting the regression analysis, all assumptions were assessed. Visual inspection of the residual plots indicated acceptable linearity and homoscedasticity. The residuals were approximately normally distributed, no evidence of multicollinearity was observed (Tolerance = 0.93, VIF = 1.08), the Durbin-Watson statistic (1.98) indicated independence of residuals, and the maximum Cook’s distance (0.078) suggested that no influential outliers were present. All sociodemographic variables were initially entered into the regression model after categorical variables were transformed into dummy variables.

The final regression model was statistically significant (F(2, 81) = 9.836, p < 0.001), explaining 26.9% of the variance in mothers’ home-management practice scores (R² = 0.269; adjusted R² = 0.245). Mothers’ knowledge and attitudes were identified as significant predictors of practice scores. Higher maternal attitude scores were significantly associated with better home-management practices (B = 1.029, β = 0.250, 95% CI: 0.106–1.952, p = 0.032). Likewise, higher knowledge scores were significantly associated with better home-management practice scores (B = 0.377, β = 0.410, 95% CI: 0.179–0.575, p < 0.001) (Table 5).

**Table 5:** Multiple linear regression analysis of predictors of mothers’ home-management practice scores (N = 84).

Predictor	B	SE	β	t	p	95% CI for B	Tolerance	VIF
						Lower – Upper		
Attitude	1.029	0.471	0.250	2.184	0.032	0.106 to 1.952	0.93	1.08
Knowledge	0.377	0.101	0.410	3.730	<0.001	0.179 to 0.575	0.93	1.08

Note. DV = mothers’ home-management practice score. B = unstandardized regression coefficient; SE = standard error; β = standardized regression coefficient; CI = confidence interval; VIF = variance inflation factor. Model statistics: F(2,81) = 9.836, p < 0.001; R² = 0.269; Adjusted R² = 0.245; Durbin–Watson = 1.98; Maximum Cook’s distance = 0.078.

## Discussion

This study investigated mothers’ home-management practices during febrile seizure episodes among children under six years of age in Jordan. Overall, approximately two-thirds of mothers demonstrated appropriate home-management practices. The mothers, however, despite having knowledge of the recommended first-aid procedures, reported a considerable number of responses that were either anxiety-driven or potentially unsafe, indicating the need for structured caregiver education and standardized post-febrile seizure counselling.

Most mothers reported placing the child in a safe position, observing the signs and duration of the seizure, and taking the child to a doctor if necessary. These findings are consistent with current international guidelines, which recommend placing the child in a safe position, monitoring seizure duration, avoiding potentially harmful interventions, and seeking medical attention when appropriate. These measures continue to be recommended in recent first-aid management reviews and remain the basis of febrile seizure management [2,17].

Most mothers took appropriate first-aid measures; however, some reported inappropriate practices, including panicking, shaking the child, restraining movement, performing cardiac massage, and placing objects in the child’s mouth. The present study did not collect information on the specific objects placed in the child’s mouth. However, previous studies have reported that caregivers used items such as fingers, spoons, cloths, or other hard objects in an attempt to prevent tongue biting. However, current clinical recommendations strongly discourage this practice because it does not prevent tongue injury and may increase the risk of oral trauma, dental injury, choking, and aspiration [18].

Similar unsafe practices have been documented in studies from Saudi Arabia, Egypt, Europe, and other regions, suggesting that misconceptions regarding febrile seizures remain common despite increasing public awareness [8,11,15].

Caregiver anxiety, often described as “fever phobia,” has consistently been associated with exaggerated responses to childhood fever and febrile seizures. Recent evidence suggests that caregiver anxiety remains common despite improvements in public awareness and is associated with increased healthcare utilization and inappropriate home-management practices [19].

In the Jordanian setting, there is limited research specifically addressing mothers’ real-time responses to febrile seizure episodes. The present study therefore provides important context-specific information on caregivers’ home-management behaviors. Understanding these behaviors is essential for developing culturally appropriate interventions to improve first-aid responses and reduce potentially unsafe management practices.

Pediatric nurses and other healthcare professionals play a critical role in enhancing caregiver preparedness for future febrile seizure episodes. Providing written discharge instructions, education about the generally benign nature of febrile seizures, and clear guidance on appropriate first-aid management may help reduce anxiety-related responses and promote safer home management. Implementing standardized caregiver education protocols in pediatric outpatient clinics and emergency departments may therefore be a practical strategy for improving home-based seizure management.

The proportion of mothers demonstrating appropriate home-management practices in the present study (68.4%) is consistent with findings reported across several Middle Eastern countries. Studies conducted in Egypt, Saudi Arabia, and several other countries have similarly reported that although many caregivers adopt appropriate first-aid measures, misconceptions and potentially unsafe responses during febrile seizure episodes remain common [8,15,20]. These similarities may reflect shared cultural beliefs, caregiver anxiety, and comparable healthcare contexts within the region. Comparable findings have also been reported in European populations, suggesting that inappropriate responses to febrile seizures remain a global challenge despite increased public awareness and improvements in healthcare services [8,11].

### Implications for clinical practice

This study highlights the need to strengthen caregiver education on the first-aid management of febrile seizures in pediatric healthcare settings. Physicians and pediatric nurses play an essential role in educating parents about safe positioning, seizure duration, and situations requiring urgent medical attention. Structured pre-discharge caregiver education is needed and should include standardized written leaflets, illustrated first-aid instructions, brief demonstration sessions, and opportunities for caregivers to ask questions. Reinforcing these messages during outpatient follow-up visits and primary healthcare visits may further improve caregivers’ confidence and reduce unsafe home-management practices during future febrile seizure episodes. These recommendations are supported by recent international evidence showing that structured caregiver education improves first-aid knowledge and reduces unnecessary emergency department visits [17,19]. The findings of this study provide useful information about mothers’ home-management practices during febrile seizure episodes. However, they should be interpreted in light of the study sample and setting and should not be considered representative of all mothers in Jordan, particularly those receiving care in rural areas, primary healthcare centers, or private healthcare settings.

### Strengths and limitations

The current study offers context-specific data on home-management practices of mothers in cases of febrile seizure in Jordan, which adds to the limited regional literature covering caregiver responses to the situation in the real-world conditions. There are, however, number of limitations to consider. First, the use of convenience sampling from two public hospitals in northern Jordan may limit the generalizability of the findings. Mothers attending tertiary public hospitals may differ from those seeking care in primary healthcare centers, private hospitals, or rural healthcare facilities with respect to healthcare access, educational opportunities, and previous exposure to health education. Therefore, the findings should be interpreted with caution and may not be representative of all Jordanian mothers. Second, mothers’ home-management practices were assessed using self-reported data collected through face-to-face interviews. As a result, the findings may be subject to recall bias, as participants may not have accurately remembered their responses during previous febrile seizure episodes, particularly if these occurred some time before the interview. In addition, social desirability bias may have influenced participants to report practices they perceived as appropriate or expected by healthcare professionals rather than their actual behaviours. Finally, because of the cross-sectional design, the observed relationships should be interpreted as associations rather than causal relationships.

## Conclusion

This study showed that although the majority of mothers reported using appropriate home-management practices during febrile seizure episodes, a considerable proportion still reported anxiety-driven or unsafe responses. Unsafe behaviors such as panicking, shaking the child, restraining movement, and placing objects in the child’s mouth remain important concerns and highlight the need for structured caregiver education. Improving mothers’ preparedness through standardized counseling and clear discharge instructions may promote safer responses during future febrile seizure episodes and reduce the risk of complications associated with inappropriate home management.
